# Stepping into adulthood: pediatric cancer survivors and their parents’ perspectives on the transition from pediatric to adult care

**DOI:** 10.1186/s12913-025-12326-3

**Published:** 2025-02-04

**Authors:** Aleshchenko Ekaterina, Langer Thorsten, Calaminus Gabriele, Glogner Juliane, Enno Swart, Katja Baust

**Affiliations:** 1 Institute of Social Medicine and Health Systems Research, Faculty of Medicine, Otto von Guericke University, Magdeburg, Germany; 2 University Hospital of Schleswig-Holstein, Campus Lübeck, Lübeck, Germany; 3 Department of Pediatric Hematology and Oncology, University Hospital Bonn, Bonn, Germany

**Keywords:** Cancer survivorship, Follow-up care, Pediatric cancer, Psycho-oncology, Self-management, Transition from pediatric to adult healthcare

## Abstract

**Aims:**

The transition from pediatric to adult healthcare is crucial for adolescent cancer survivors due to the potential lifelong late effects of their treatment. Despite the importance of ongoing follow-up care, attendance often declines after transitioning to adult services. This study explores the perspectives of adolescent and adult pediatric cancer survivors and their families on this transition process.

**Methods:**

Using the Theory of Planned Behaviour, we developed interview guidelines and conducted 36 episodic narrative interviews with survivors and their parents. Our analysis focuses on their transition experiences and expectations, as part of the broader VersKiK-Study aimed at improving (long-term) follow-up care for pediatric cancer survivors in Germany.

**Results:**

Findings show that although transitioning is viewed as a significant step toward adulthood, a lack of preparedness and anxiety can hinder a shift between healthcare systems. Survivors expressed the need to initiate the transition process while still under pediatric care. Clear and effective communication was identified as key to ensuring a smooth transition. Adult survivors acknowledged the transition's importance in fostering self-reliance and independence.

**Conclusions:**

To facilitate better outcomes, transition planning should include early introductions to adult care providers and strong communication strategies to address the emotional and psychosocial challenges faced by adolescent cancer survivors. Aligning transition practices with the needs and concerns of survivors could enhance follow-up care engagement, ultimately improving long-term outcomes for pediatric cancer survivors.

## Background

Being mostly treatable, cancer in childhood or adolescence may often result in life-long late effects. The objective of (long-term) follow-up care is to minimize their impact through prevention, early detection, or effective treatment, aiming to enhance survivor’s overall quality of life [1]. However, many of them tend to stop attending regular follow-up appointments once they transition from pediatric care, with a gradual decline in follow-up over time after completing their treatment [2, 3].

The transition from pediatric to adult-centered healthcare facilities for adolescents who have undergone cancer treatment is a complex process involving developmental, health-related, and organizational changes, such as a control perception, lack of confidence in self-management capabilities, or issues with autonomy from parents [4]. This multifaceted process requires careful consideration of personal preexisting factors such as age, gender, emotional and social maturity, health status, and situational contexts, involving their parents, and healthcare providers [5].

Naturally, parents play active roles in the survivor’s lives and are usually active in managing their health conditions. Parents serve as informal care managers, taking on responsibilities to support young survivors as they navigate the challenges of becoming independent [6]. Even as young people progress towards independence in managing their health conditions, some may still seek guidance from their parents regarding their care [7]. Parents express worries about their children transitioning from pediatric to adult care, particularly regarding their ability to advocate for themselves, manage their illness, and receive appropriate care as they enter adulthood [8]. Parents may feel ambivalent about the transfer and may hesitate to relinquish their roles in their children's care [9]. The majority of parents expect their adolescents to self-manage their medical care after that transfer, although they are concerned about potentially reducing their involvement in this process [10]. Moreover, parents aim to strike a balance between relinquishing control and maintaining some level of oversight in their children's medical care [11]. The latter is often dependent on the subjective perception of the “right timing” for the transition, - a time point, when survivors feel comfortable and prepared for the process [12, 13].

The roles of different healthcare providers, directly involved in (long-term) follow-up care, vary depending on several factors. Firstly, the most common obstacle in transitioning to adult healthcare facilities is the lack of knowledge among primary care practitioners about the long-term effects of pediatric cancer (e.g. [14, 15]). Additionally, a perceived emotional attachment to pediatric oncologists makes the psychological aspect of transitioning to adult medicine challenging [16].

In Germany, the transition from pediatric to adult healthcare lacks a standardized approach and is often left to the initiative of individual doctors or hospitals, as well as the active involvement of patients' families [17]. This process is complicated by the structural separation between pediatric and adult healthcare systems, which typically do not have mechanisms for direct information exchange. Thus, up to 40% of adolescent patients lose access to appropriate specialist care during the transition from pediatric and adolescent medicine to adult medicine [18, 19]. Recently, specialized guidelines have been developed to support this transition, aiming to address its complexity and improve outcomes [20] and currently funded health care studies address this gap [21]. However, implementing these guidelines remains challenging due to variations in local healthcare practices, resource availability, and the need for alignment across diverse stakeholders.

Different evidence-based tools and models were suggested to tackle the challenge of addressing complex and dynamic issues related to transition. SMART model, the Social-ecological Model of Readiness for Transition, developed specifically for use with adolescents and young adults with chronic health conditions, was already adopted for pediatric cancer survivors [22, 23]. This framework examines barriers and facilitators across multiple system levels, providing a comprehensive perspective on the transition process. It integrates individual survivor factors—such as developmental readiness, knowledge, and self-efficacy—with interconnected elements, including relationships with parents and healthcare providers, as well as broader psychosocial aspects. For successful practical application, it is critical to incorporate the different perspectives and coping strategies of survivors and their parents.

To address this gap, this study aimed to investigate the experiences and expectations of pediatric cancer survivors, both adolescents and adults, as well as the perspectives of their parents regarding the transition from pediatric to adult healthcare facilities.

## Methods

This analysis is part of the larger VersKiK project, which investigates the long-term effects of childhood and adolescent cancer, adherence to specific long-term follow-up guidelines, and the actual long-term follow-up needs of survivors and their parents. The overall design of the VersKiK project and a detailed description of this study's methodology are provided in separate publications [24, 25].

The VersKiK project was approved by the Ethics Committee Otto von Guericke University on 2.07.2021 (103/21), by the Ethics Committee of Johannes Gutenberg University Mainz on 16.06.2021 (2021–16035), by the Ethics Committee University of Lübeck on 10.11.2021 (21–451), by the Ethics Committee University of Hospital Bonn on 28.02.2022 (05/22). For each part of the qualitative study, the Ethics Committees approved a separate written informed consent.

### Design

To investigate the perspectives of survivors and their parents on the transition process, we created episodic narrative interview guidelines in German [26]. These guidelines were adopted for adolescent (12–18 years old) and adult survivors and their parents , - e.g., using appropriate language and minor changes to the questions. The interview guideline had a modular structure, aiming to address dimensions of Theory of Planned Behaviour [27] The comprehensive process of developing the interview guideline is described elsewhere [28].

This article analyzes a specific set of questions focused on transition issues. To capture diverse perspectives on the transition process, all interview participants were asked these questions, regardless of their or their children's transition status (whether it had not started, was in progress, completed, or not undertaken). The only variation in the questions was based on the participant's current age and how close they were to the transition period. The interview guidelines are outlined in Table 1.

**Table 1:** Interview guidelines

**Jugendliche**	**Adolescents**
1. Was verstehst Du unter dem Wechsel von der Kinderonkologie zu den Erwachsenen-Ärzt*innen (Transition)? Likert: Ich fühle mich bereit für die Transition. Wenn untere Hälfte der Skala: Warum fühlst Du Dich nicht bereit für die Transition? Wenn obere Hälfte der Skala: Welche Aufgaben siehst Du für Dich im Zusammenhang mit der Transition? Zusätzliche Frage: Würdest Du gern selbst entscheiden, wann Deine Transition beginnt?	1. What do you understand by the transfer from pediatric oncology to adult doctors (transition)? Likert: I feel ready for the transition. If the lower half of the scale: Why don't you feel ready for a transition? If the upper half of the scale: What tasks do you see for yourself concerning the transition? Additional question: Would you like to decide by yourself when your transition begins?
2. Fühlst Du dich wohl, wenn Du als erwachsen behandelt wirst? Likert: In welchem Ausmaß fühlst Du Dich in Deinen Nachsorge-Prozess eingebunden? Wenn untere Hälfte der Skala: Warum fühlst Du Dich nicht genügend in den Prozess eingebunden? Wenn obere Hälfte der Skala: Was trägt dazu bei, dass Du Dich eingebunden fühlst?	2. Do you feel comfortable being treated as an adult? Likert: To what extent do you feel involved in your follow-up care process? If the lower half of the scale: Why don't you feel involved enough in the process? If the upper half of the scale: What makes you feel involved?
3. Wer organisiert Deine Nachsorgetermine? Likert: Ich bin bereit, meine Nachsorge selbst zu planen. Wenn untere Hälfte der Skala: Warum fällt es Dir schwer? Wenn obere Hälfte der Skala: Was gibt Dir Zuversicht, es selbst zu schaffen?	3. Who organizes your follow-up appointments? Likert: I am prepared to organize my follow-up care myself. If the lower half of the scale: Why do you find it difficult? If the upper half of the scale: What gives you the confidence to do it yourself?
4. Denkst Du, dass Du auch noch ein Nachsorge-Angebot brauchst, wenn Du erwachsen bist? Likert: Wie zuversichtlich bist Du, an Deinem nächsten Nachsorgetermin teilzunehmen? Wenn untere Hälfte der Skala: Welche Art Unterstützung bräuchte es, damit Du regelmäßig an Deinen Nachsorgeterminen teilnehmen kannst? Wenn obere Hälfte der Skala: Was oder wer sorgt dafür, dass es Dir eher leichtfällt, an regelmäßigen Nachsorgetermine teilzunehmen?	4. Do you think you will still need follow-up care when you are an adult? Likert: How confident are you about attending your next follow-up appointment? If the lower half of the scale: What kind of support would it take for you to attend your follow-up appointments regularly? If the upper half of the scale: What or who makes it rather easy for you to attend regular follow-up appointments?
**Sorgeberechtigte**	**Informal careers**
1. Was verstehen Sie unter dem Übergang von der Kinderonkologie zu den Erwachsenen-Ärzt*innen (Transition)? Likert: Die Transition kommt für mein Kind zum richtigen Zeitpunkt. Wenn untere Hälfte der Skala: Welche Hürden bestehen Ihrer Meinung nach? Wenn obere Hälfte der Skala: Wieso ist es ein guter Zeitpunkt? Wenn Ihr Kind ein gesundheitliches Problem hat, bei dem Sie denken, dass es sich um eine Spätfolge handeln könnte, wer ist Ihr erster Ansprechpartner? Weshalb ist dies in der Regel ihr erster Ansprechpartner?	1. What do you understand by the transition from pediatric oncology to adult doctors? Likert: The transition comes at the right time for my child. If the lower half of the scale: What hurdles do you think exist? If the upper half of the scale: Why is it a good time? If your child has a health problem that you think could be a late effect, who is your first point of contact? Why is this usually your first point of contact?
2. Welche Gefühle haben Sie, wenn Sie an die Transition Ihres Kindes denken? Likert: Ich bin bereit meinem Kind die Verantwortung für seine/ihre eigene Nachsorge zu übergeben. Wenn untere Hälfte der Skala: Warum fällt es Ihnen eher schwer? Wenn obere Hälfte der Skala: Wie ermutigen Sie ihr Kind, selbst mehr Verantwortung zu übernehmen?	2. What feelings do you have when you think about your child's transition? Likert: I am ready to give my child responsibility for his/her follow-up care. If the lower half of the scale: Why do you find it rather difficult? If the upper half of the scale: How do you encourage your child to take on more responsibility themselves?
3. Welche Aufgaben sehen Sie für sich bei der Transition Ihres Kindes? Likert: Die Transition findet zu einem guten Zeitpunkt statt. Wenn untere Hälfte der Skala: Was könnte für Sie ein Merkmal des „richtigen Zeitpunkts“ sein? Wenn obere Hälfte der Skala: Was gibt Ihnen Zuversicht, dass Ihr Kind die Transition selber steuern kann?	3. What tasks do you see for yourself in your child's transition? Likert: The transition is taking place at a good time. If the lower half of the scale: What could be a characteristic of "the right time" for you? If the upper half of the scale: What gives you confidence that your child can manage the transition themselves?
**Erwachsene**	**Adult Survivors**
1. Welche Erfahrungen haben Sie mit Ihrem Wechsel von der Kinderonkologie zu den Erwachsenen-Ärzt*innen (Transition) gemacht? Likert: Ich bin mit meiner Transition zufrieden (gewesen). Wenn untere Hälfte der Skala: Was ist, Ihrer Meinung nach, nicht so gut gelaufen? Bitte nennen Sie ein paar Beispiele. Wenn obere Hälfte der Skala: Was war dabei gut? Bitte nennen Sie ein paar Beispiele.	1. What experiences have you had with your transfer from pediatric oncology to adult doctors (transition)? Likert: I (was) satisfied with my transition. If the lower half of the scale: In your opinion, what did not go so well? Please give a few examples. If the upper half of the scale: What was good about it? Please give a few examples.
2. Hat sich seit dem Wechsel in die Erwachsenenmedizin etwas an Ihrer medizinischen Versorgung verändert? Likert: Die Transition hat signifikante Veränderungen meiner Versorgung gebracht. Wenn untere Hälfte der Skala: Was hat Ihnen geholfen, reibungslos durch diesen Prozess zu kommen? Wenn obere Hälfte der Skala: In welchen Bereichen war diese Veränderung am stärksten? Bitte nennen Sie ein paar Beispiele.	2. Has anything changed in your medical care since the transition to adult medicine? Likert: The transition has brought significant changes to my care. If the lower half of the scale: What has helped you get through this process smoothly? If the upper half of the scale: In which areas was this change most significant? Please give a few examples.
3. Wie stellen Sie sich die Organisation einer idealen Nachsorge vor? Likert: Mein Hausarzt/Meine Hausärztin geht auf meine spezifischen Nachsorge-Anliegen (Untersuchungen, Fachspezialisten) ein. Wenn untere Hälfte der Skala: Welche Schwierigkeiten hat das für Sie schon verursacht? Nennen Sie bitte ein paar Beispiele. Wenn obere Hälfte der Skala: Wie unterstützt Sie Ihr Hausarzt/Ihre Hausärztin konkret?	3. How do you picture the organization of ideal follow-up care? Likert: My family doctor addresses my specific follow-up care concerns (examinations, specialists). If the lower half of the scale: What difficulties has this already caused for you? Please give a few examples. If the upper half of the scale: How specifically does your family doctor support you?

### Recruitment

Participants were recruited using a convenience sampling method [29], with participation based on a volunteer basis, requiring individuals to invite themselves. Recruitment was carried out through specialized media outlets such as WIR, a German magazine aimed at childhood cancer patients, survivors, and their parents, and social media platforms managed by the Children's Cancer Foundation^1^. Additionally, participating hospitals were involved to ensure a diverse range of participants in terms of age, cancer diagnoses, and (long-term) follow-up duration. Eligibility criteria included individuals regardless of their type of treatment received, cancer diagnosis, stage, or grade of cancer survived. The sole exclusion criterion was an insufficient cognitive function that could potentially hinder participation in the interview.

### Data collection

EA and JG conducted 36 interviews across multiple locations: the University Hospital Schleswig-Holstein and the University Hospital Bonn, both of which have specialized multidisciplinary long-term follow-up care, and the University Hospital Magdeburg, which lacks such facilities. These interviews took place between September 2022 and March 2023. Because of the COVID-19 pandemic, one interview was conducted via video call. We obtained written informed consent from all participants, and for minors, we also collected the signature of a parent .

### Analyses

We employed Hsieh and Shannon's methodology to apply content analyses for the interview transcripts, following a structured five-step approach [30]. Initially, the process involved transforming textual data into narrative form and identifying units of analysis and key themes. Coding rules were established to systematically apply the coding system across all narrative data. This coding system was continuously refined as needed to ensure comprehensive coverage of significant concepts and recurring themes within the narratives. The thematic coding process allowed us to systematically classify and categorize the main themes identified in the interviews. Themes were initially defined by EA and then reviewed by KB to ensure consistency and accuracy in capturing the essence of the messages conveyed in the data.

We conducted this study following COREQ guidelines [31] and declaration of Helsinki [32].

## Results

The general characteristics of interviewees are presented in Table 2 “General characteristics of interviewees”.

**Table 2:** General characteristics of interviewees

	Year of birth	Diagnosis	Year since Diagnoses	Transition patient (18–25 years old)	Adult patient	Adolescent	Informal caregiver	Relapse
1	1996	Suprasellar germinoma	6.8		X			N
2	1992	HL	1.7		X			N
3	1993	Rhabdomyosarcoma	18.1		X			N
4	2000	Osteosarcoma	13.2	X				N
5	2006	Osteosarcoma	4.3	X				N
6	2006	HL	1.5	X				N
7	2005	Osteosarcoma	2.6	X				N
8	2002	Ewing's sarcoma	4.0	X				N
9	2003	ALL	6.0	X				N
10	2004	Nephroblastoma	10.4	X				N
11	2006	Osteosarcoma	4.3				X	N
12	2006	HL	1.5				X	N
13	2005	Osteosarcoma	2.6				X	N
14	2002	Ewing's sarcoma	4.0				X	N
15	2003	ALL	6.0				X	N
16	2004	Nephroblastoma	10.4				X	N
17	2001	Neuroblastoma	3.7		X			N
18	1982	ALL	37.6		X			N
19	2000	NET	20.1		X			N
20	2003	Brain tumour	6.7		X			N
21	2000	Brain tumour	20.1		X			Y
22	1988	HL	21.7		X			N
23	1967	Leukaemia	17.8		X			Y
24	2004	Rhabdomyosarcoma	14.2			X		N
25	2006	Neuroblastoma	14.7			X		N
26	2003	Brain tumour	5.6				X	N
27	2003	Brain tumour	11.6				X	N
28	2004	Rhabdomyosarcoma	14.2				X	N
29	2003	Neuroblastom	5.6	X				Y
30	1978	HL	17.3	X				N
31	2004	CML	5.7	X				N
32	2010	ALL	3.7			X		N
33	2008	ALL	2.7			X		N
34	2004	CML	5.7				X	N
35	2008	ALL	2.7				X	N
36	2010	ALL	3.7				X	N

Diagnosis: *HL* Hodgkin's lymphoma, *NHL* Non-Hodgkin lymphoma, *CML* Chronic myeloid leukemia, *ALL* Acute lymphoblastic leukemia, *NET* Neuroendocrine tumor, *Y* yes, *N* no

Table 3 summarizes the perspectives of adolescent and adult survivors, and their parents on transitioning from pediatric to adult care. Further, we outline the most detail and provide more context for their understanding.

**Table 3:** General themes from Interviews

	Adolescents	Informal Careers	Adult Survivors
Definition of transition	Define transition mainly as a time when they have to take on more responsibility. It is associated with growing up and the accompanying duties such as working and earning money.	See transition as a change of organizational form and type of care. It is emphasized that it is also about young adults learning to manage their health independently.	See transition as a change in the form of care and organizational structure. It is seen as a formal change from pediatric oncology to adult medicine, which is necessary because the billing system requires it.
The “right time”	Mostly are not yet feeling ready for the transition and would prefer to decide for themselves when the time is right, often with a tendency to delay this.	Are often of the opinion that their children are not yet ready and are worried about the transition. They want to ensure that their children are well prepared and have the necessary skills to act independently.	Consider the 18th birthday to be an appropriate time for the transition, as this coincides with other aspects of growing up, such as organising oneself and taking on responsibility.
Changes in healthcare through the transition	See the transition as a phase with more duties and responsibilities, as well as a challenge because of need to get used to new doctors.	Have concerns about the quality of care in adult medicine and their children's ability to take on the new responsibilities.	See the transition mainly as a formal change in care and organisation, but also recognise that more responsibility is transferred to the patient.
Responsibility for the organisation of care	Perceive taking on responsibility as a major challenge and would often prefer to postpone it.	Emphasise the importance of gradually transferring responsibility so that their children feel safe and can develop confidence in their own abilities.	See it as an important step for young adults to learn to manage their own healthcare. They see the transition as part of the process of preparing their children for adult life.

### Definition of transition

Interviewees from different groups emphasized various aspects when defining the concept of "transition." Adolescents mainly see transition as an integral part of maturing and entering adulthood, accompanied by tangible responsibilities like employment and financial independence. They put more focus on the personal growth aspect, viewing transition as a time to become more self-reliant.

“More responsibility. At some point, you move, get married, and have kids. But even before that, you might move out and live alone or with someone else, and you have responsibilities. You have to earn money and work. So, yeah, there are these duties you have to take care of.” (Adolescent survivor, 14,7 years after diagnoses)

Parents see transition as a change in organizational form and type of care. This perspective underscores the importance of survivors learning to manage their health independently. Similarly, adult survivors reflect on the transition as essential for the continuity and appropriateness of care within the healthcare framework. Transition is mostly seen as necessary to align with new adult tasks in all spheres of life .

“I think it's because I've taken responsibility for myself in other areas too. I don't live at home anymore, I have my job, and so on. That's why it felt right. I was confident enough to say that I'd handle this follow-up examination on my own.” (Adult survivor, 3,7 years after diagnoses)

### “The right time” for the transition

The perception of the "right time" for transition also varies among adolescents, parents, and adult survivors, reflecting their differing roles and experiences.

Adolescents often feel unprepared for the transition and prefer to delay it until they feel psychologically ready. They wish to have the autonomy to decide when to make the shift, often postponing it to avoid the challenges and responsibilities that come with the transition.

“I’d want to figure it out for myself, maybe through a bit of discussion or something like that. I’d like to hear the pros and cons and understand why it might be important from a medical standpoint. At the end of the day, the doctors have the final say, and if something’s really important, I’d go along with it. But I’d still want to understand what’s going on and share my own thoughts.” (Transition survivor, 1,5 years after diagnoses)

Parents share similar concerns about readiness but from a protective perspective. They frequently believe that their children are not yet psychologically prepared for the transition and worry about their ability to manage independently.

“I think she needs a bit more time, for both her and us. We’re not quite ready to let go completely yet. With our daughter, there are still some understanding issues—she’s still really dependent and doesn’t quite get it.” (Parent of survivor 14,2 years after diagnoses)

In contrast, adult survivors often view the 18th birthday as an appropriate time for transition. They see this milestone as a natural time to take on adult responsibilities, in line with other life changes that happen at this age, bringing greater independence and self-management.

### Changes in healthcare through the transition

Adolescents perceive the main change as increase in duties and responsibilities. They are particularly apprehensive about adapting to new doctors and unfamiliar medical environments, which adds to the challenge of managing their health independently.

“You already know your current doctor and understand each other well. You’re familiar with the contacts and everything. But if you switch to an adult clinic, you’ll be dealing with new faces—new nurses and doctors who won’t know the details of your treatment. Only the pediatricians will have that info. That’s why I’d prefer to stay where we are” (Adolescent survivor, 2,7 years after diagnoses)

Parents, on the other hand, express significant concerns about the quality of healthcare in adult medicine. They worry about whether their children will receive the same level of attention and support they had in pediatric healthcare.

‘‘How do I feel about it? Well, in pediatric care one can rely on the ward staff to do thorough examinations and great availabilities. Expecting long waiting times and fewer available appointments….let’s just say it makes me feel rather pessimistic.“(Parent of survivor 2,7 years after diagnoses)

Adult survivors typically see the transition primarily as a formal change in organizational structure. They recognize that the transition involves shifting from pediatric to adult medicine, which is largely driven by administrative and healthcare system-related requirements. However, they also acknowledge that this change transfers more responsibility to the patient, who must now take a more active role in managing their health.

“Yes, I think the best part was getting more information and being told more about everything. It’s probably because, in the pediatric ward, some kids might not be able to communicate about things very well. When you switch to the adult clinic, you get talked to more directly and not just through your parents. So, getting more personal attention and detailed information was the best part.” (Transition survivor, 13,2 years after diagnoses)

### Responsibility for the organization of care

Adolescent interviewees expressed both readiness and anxiety about taking full responsibility for their health care. They understood the necessity of the transition but felt uncertain about their preparedness, focusing on the emotional challenges of the transition, such as the potential lack of familiar and supportive relationships with new healthcare providers in an adult healthcare setting. They fear being less well-understood by healthcare providers for adults and the possibility of feeling out of place among older patients.

“I think the familiarity part might be harder. As I said, it can make it tougher to talk about my problems. It could be really annoying to keep explaining how my health is progressing because the outpatient clinic already knows me and my case, so I don't have to repeat everything. But in adult oncology, I imagine that might happen a lot. From my experience with bone infarctions, going to different doctors over and over is just a real hassle” (Transition survivor, 6,0 years after diagnoses)

Similarly, adult survivors recalled a lack of preparation provided by the involved institutions and support during their transition period. They often felt abandoned when moving to adult care services, so they stressed the importance of emotional and psychological support during the transition. They also noted that having a mentor or a transitional coordinator would have been beneficial. The need for comprehensive access to medical histories and improved communication tools, such as digital applications, was highlighted as essential and should be provided by overseeing healthcare professionals, such as general practitioners or oncologists.

“That was a big turning point for me. At some point, I was told I was too old—I think I was around 19, 20, or 21. I can't remember exactly. I guess I didn't fit into the usual pediatric clinic anymore. But it would have been nice to keep seeing the same doctors I'd had for years. In the end, I was just too old, so I had to go somewhere else and find new doctors. And those doctors seemed to change every two years.” (Adult survivor, 18,1 years after diagnoses)

Similarly, parents emphasize the importance of transferring responsibility gradually to their children. They believe that this approach helps their children feel safe and secure while building confidence in their ability to manage their healthcare over time.

“He’s going to have to take on more personal responsibility, like keeping track of his appointments. … Right now, he’s always with me and at home, but once he’s out on his own, he’ll need to handle a lot more on his own. It’s not just about these appointments, but responsibility in all areas of his life.” (Parent of survivor 2,6 years after diagnoses)

They often see their role as ongoing supporters, ensuring their children remain attentive to their health. They recognize the need for a balance between supporting their child and allowing them to become independent.

“I see it as my job as a mom to help her feel ready to go on her own next year. This year she said, 'Mom, I'm 18, but I'm not going alone. You're coming with me.' But from the individual conversations she had without me during the appointment, she's learned that she can trust herself to go on her own.” (Parent of survivor 6,7 years after diagnoses)

## Discussion

This study explored the transition experiences and expectations of survivors and their parents. The findings align with the Social-Ecological Model of Readiness for Transition (SMART), which highlights multiple system-level barriers and facilitators influencing the transition process. These include individual survivor factors such as developmental maturity, knowledge, and self-efficacy, as well as interconnected elements involving parents and healthcare providers, such as expectations, relationships, and psychosocial functioning parents [22]. Interconnected constructs are particularly promising as targets for interventions. Similarly, a systematic review of 15 studies conducted by Otth et al. [33] similarly defined the following factors affecting the transition experiences of pediatric cancer survivors: self-management skills, social environment, subjective feelings, pediatric/adult setting, financial issues and insurance, communication, and structural circumstances including organization of transition.

*Developmental maturity* was addressed in our research through readiness for the transition. Adolescent survivors generally felt unprepared for the transition and expressed a desire to choose the “right time” to begin attending adult healthcare facilities on their own. Parents shared a similar view, wanting to ensure that young survivors had developed *self-efficacy* skills to manage their follow-up care before transitioning. These findings align with those of previous research indicating that self-efficacy skills are crucial facilitators in the transition process [33, 34]. Interestingly, adult survivors in our study retrospectively viewed the age-defined timing for transition as appropriate, likely due to the self-management skills and knowledge they developed over time while dealing with late effects on their own.

Previous research has identified *knowledge* deficits about the disease and a lack of recognition of the benefits of transition as primary barriers to a successful transition (e.g. [5, 33, 35]). In our study, parents acknowledged the need to gradually "empower" young survivors by increasing their understanding of their health condition and emphasizing the importance of (long-term) follow-up care.

Within the SMART framework, *expectations* are defined as a survivor's trust in adult healthcare facilities and the importance of long-term follow-up care. While adolescent survivors in our study generally understood the significance of long-term follow-up due to their parents' explanations, they expressed considerable doubts about trusting adult healthcare facilities. These findings align with previous research, which indicates that survivors' attachment to pediatric healthcare facilities can hinder the transition and negatively impact their trust in the quality of care provided by adult healthcare professionals (e.g. [14, 33]). Adult survivors in our study also noted that developing self-management skills by handling their follow-up care independently contributed to their overall maturity. However, they felt that emotional and psychological support during the transition would have been highly beneficial.

One of the primary *goals* within the SMART framework for the transition process is to facilitate survivors' autonomy. Previous research has shown that adolescents who were not actively involved in their healthcare became dependent on their parents' participation, leading to a reliance on others for help [4, 36]. This dependency fostered a passive role that continued into adulthood, with parents still heavily involved in communicating with healthcare services as their children matured. In our study, parents recognized the necessity of gradually transferring responsibility for healthcare and interactions with providers to the survivors. They also emphasized the need for information to be presented as child-friendly to aid this transition. This aspect is closely linked to the *relationships among survivors, parents, and healthcare providers*. Previous studies have indicated that parents experience "cross-pressure" [6], balancing their child's and healthcare providers' expectations while supporting their young adult's transition to independence. This might be caused by complexities in building relationships with new adult healthcare providers. Building a new relationship requires time and usually cannot be achieved in just one consultation hour. Consequently, survivors prefer to initiate this new relationship with adult providers toward the end of their pediatric care while they are still within the pediatric environment. Effective communication between survivors, healthcare providers, and parents is also a crucial factor during the transition from pediatric to adult care [19, 33].

Effective communication also contributes to enhanced *psychosocial functioning*, which the SMART framework interprets as emotions related to the transition process. Adolescent survivors interviewed perceived this transition as a step into adulthood and a sign of maturity, leading many of them to express concerns about their readiness. They worry about transitioning to unfamiliar adult healthcare providers and feel discouraged by the need to repeatedly recount their cancer experiences, which may bring up potentially traumatic memories. This aligns with previous research, which suggests that young adult survivors' desire to move beyond their cancer experience can influence their participation in regular follow-up appointments [19, 37]. Likewise, adult survivors, reflecting on their various transition experiences, view it as a crucial process that teaches self-reliance and independence [38].

## Implications

Our findings emphasize the need for a structured and multidisciplinary approach to enhance the transition process for pediatric cancer survivors in Germany. Early preparation is crucial, including tailored self-management training to empower survivors and gradual responsibility transfer from parents. Collaborative efforts between pediatric and adult providers are essential to ensure continuity of care, with early introductions to new providers during pediatric follow-up to foster trust and reduce worries and restraints related to the transition process.

Existing specialized multidisciplinary long-term follow-up care in Germany should be expanded to improve accessibility, enabling more survivors to benefit from coordinated care. These clinics, alongside digital tools for tracking medical histories and appointments, can facilitate a smoother transition. Survivors' emotional and psychological concerns related to the transition process such as worries about new environments and recounting medical histories as well as a relevant number of mental health problems in this group [39], highlight the importance of transition coordinators and psychologists as part of the multidisciplinary team These findings underscore the importance of psychological readiness and the inclusion of survivors' and parents' perspectives, further aligning with specialized guidelines' recommendations for organizing transitions and ensuring comprehensive, patient-centered support [20].

Building on frameworks like SMART, interventions should address readiness, communication, and self-efficacy to align survivor experiences with system demands. By enhancing these aspects, healthcare systems can promote autonomy, sustain engagement in follow-up care, and improve long-term outcomes for survivors.

## Strengths and limitations

Our research expands upon existing knowledge by investigating perspectives from both adolescent and adult survivors, as well as theirparents. This dual focus allows for a more comprehensive understanding of the transition from pediatric to adult healthcare. One of the significant strengths of our study is the involvement of a multidisciplinary team of researchers. This team includes experts in healthcare research, medical professionals, and clinical psychologists, each contributing their specialized knowledge and insights. This collaborative approach enriches the study's depth and breadth, ensuring a more nuanced and well-rounded analysis of the challenges and needs associated with the transition process. By integrating diverse expertise, our research not only highlights the varied experiences and expectations of survivors and their parents but also provides a robust foundation for developing targeted interventions to support this critical transition.

We also recognize several limitations in our study. First, participants might have felt constrained in sharing negative information, potentially leading to socially acceptable responses. To mitigate this, the interview guidelines included a variety of question types. Second, the interviews were conducted on a volunteer basis, primarily in hospitals with established long-term follow-up care structures. Therefore, the perspectives collected may not fully represent pediatric cancer survivors who are unaware of the late effects of cancer in childhood and adolescence or lack knowledge of (long-term) follow-up care, and, consequently, are not actively involved in (long-term) follow-up. Additionally, this could influence responses, as survivors might fear harming their relationships with follow-up healthcare providers by giving "undesirable" answers. Thus, this limitation may exclude important perspectives on the barriers to transition and emphasizes the need for future research to incorporate a broader range of participants. Lastly, survivors may not have been ready to fully disclose potential traumatic experiences related to cancer or may still face stigma associated with their condition, and their responses could be constrained by the time elapsed since the transition and memory effects, particularly among older survivors. Moreover, responses could be affected by recall bias, particularly among participants for whom a significant amount of time had passed since their cancer diagnoses, potentially limiting the accuracy and detail of their recollections.

## Conclusion

Our study highlights the critical importance of a well-planned transition from pediatric to adult healthcare for adolescent cancer survivors and their families. Key findings reveal that the transition is perceived as a significant step towards adulthood but is accompanied by concerns regarding readiness and the anxiety of engaging with new healthcare providers.

Survivors and their families expressed a preference for starting the transition process early to build trust and familiarity with adult providers, thus reducing anxiety and improving engagement in follow-up care. Aligning to the SMART framework our results underscore the emotional and psychosocial dimensions of the transition, identifying the need for strategies that support survivors' self-reliance and independence. By addressing these factors, healthcare systems can improve long-term outcomes for adolescent cancer survivors, facilitating a smoother and more effective transition to adult care.

## Data Availability

The anonymised datasets analysed during the current study are available from the corresponding author on reasonable request.

## Footnotes

1 https://www.kinderkrebsstiftung.de/
